# Response Rate to the Intervention with Tirbanibulin 1% Ointment for Treating Actinic Keratoses in People Living with HIV Infection

**DOI:** 10.3390/diagnostics15040401

**Published:** 2025-02-07

**Authors:** Giulia Ciccarese, Francesco Drago, Lucia Lospalluti, Mauro Grandolfo, Sergio Lo Caputo, Mario Mastrolonardo, Benedetta Tirone, Cosimo Castronovi, Riccardo Bortone, Gerardo Cazzato, Caterina Foti

**Affiliations:** 1 Unit of Dermatology, Department of Medical and Surgical Sciences, University of Foggia, Viale Pinto, 1, 71122 Foggia, Italy; mario.mastrolonardo@unifg.it; 2 Casa di Cura Villa Montallegro, Via Monte Zovetto 27, 16100 Genoa, Italy; francescodrago007@gmail.com; 3 Section of Dermatology and Venereology, Department of Precision and Regenerative Medicine and Ionian Area (DiMePRe-J), University of Bari “Aldo Moro”, 70126 Bari, Italy; lucia.lospalluti@policlinico.ba.it (L.L.); grandolfo1@alice.it (M.G.); benedetta.ti96@gmail.com (B.T.); cosimocastronovi90@gmail.com (C.C.); riccardobort@gmail.com (R.B.); caterina.foti@uniba.it (C.F.); 4 Clinic of Infectious Diseases, Department of Clinical and Surgical Sciences, University of Foggia, 71122 Foggia, Italy; sergio.locaputo@unifg.it; 5 Section of Molecular Pathology, Department of Precision and Regenerative Medicine and Ionian Area (DiMePRe-J), University of Bari “Aldo Moro”, 70124 Bari, Italy; gerardo.cazzato@uniba.it

**Keywords:** actinic keratosis, tirbanibulin 1% ointment, people living with HIV

## Abstract

**Background/Objectives**: People living with HIV (PLWH) are more susceptible than immunocompetent people to non-melanoma skin cancers. These tumors can arise de novo or from precancerous lesions, such as actinic keratosis (AKs). The management of AKs in PLWH has not been widely discussed in the literature. More specifically, the efficacy of the treatment of AKs in PLWH with modern topical drugs, such as tirbanibulin, is limited. The present work aims to evaluate the response rate to the intervention with tirbanibulin 1% ointment for treating AKs in PLWH. **Methods**: We retrospectively collected the data of the PLWH who visited the Dermatology Department of the Policlinico Riuniti (Foggia, Italy) between September 2023 and September 2024. PLWH who received the diagnosis of AKs and underwent treatment with tirbanibulin 1% ointment were studied. To assess the severity of AKs, the number of AKs and the AKs’ area and severity index (AKASI) score were calculated at the time of diagnosis (T0) and after treatment (T1). **Results**: Ten PLWH were found to have AKs and received topical therapy with tirbanibulin 1% ointment. On average, at T0, the number of lesions was 8.2 and the AKASI score was 4.20; at T1, the number of AKs was 1.7 and the AKASI score was 1.5. Only two patients reported a mild inflammatory reaction to applying tirbanibulin 1% ointment. **Conclusions**: The rate of satisfactory responses was in line with a recent multicentric Italian study performed on immunocompetent patients. Our results confirm the efficacy and tolerability of tirbanibulin 1% ointment in treating AKs in PLWH in particular.

## 1. Introduction

Actinic keratosis (AK) is a cutaneous lesion that typically begins to appear in the fourth and fifth decades of age, increasing in number over the years. Clinically, an AK appears as a poorly circumscribed erythematous flat macule or a variably rough or scaly papule usually arising in the photo-exposed areas. Histologically, it is characterized by foci of atypical, pleomorphic keratinocytes confined to the lower third of the epidermal skin layer and budding into the upper papillary dermis. AK is considered either a precancerous lesion that may progress to an invasive squamous cell carcinoma (SCC) or an in situ SCC (intraepidermal proliferation of atypical keratinocytes) that may progress to an invasive stage [1,2]. Indeed, AK is cytologically indistinguishable from in situ SCC and has several molecular features ascribed to SCC [3,4].

Excessive chronic exposure to ultraviolet (UV) radiation is the main environmental factor leading to AK and SCC development. Indeed, AKs are located mainly in areas with chronically sun-damaged skin, such as the face, scalp, head and neck area, and hands [5]. Mutations and the deregulation of tumor suppressor proteins such as p53, p16INK4a, and PTEN induced by UVB radiation are considered crucial pathogenetic mechanisms in the development of AK and SCC [5,6,7].

Concerning epidemiology, the prevalence of AK varies significantly across countries, depending on the study setting, UV radiation level and patient characteristics. In Australia, up to 60% of people over 40 have AKs, while in Italy, the prevalence is estimated at 27.4% [2].

Factors associated with an increased risk of AK in immunocompetent individuals are: male sex, age > 45 years, fair skin type, light hair and eye color, the presence of freckles on face and arms, a positive history of non-melanoma skin cancer (NMSC), repeated sunburns occurred in childhood and adulthood, chronic occupational and/or recreational sun exposure, severe baldness, and the use of potentially photosensitizing drugs such as thiazide diuretics or other cardiac drugs [2,8]. Conversely, factors associated with a reduced risk of AK are regular sunscreen use and, probably, a history of atopy [2,9,10,11].

The diagnosis of AK is usually made by clinical examination. Individual AK lesions have been clinically graded based on their thickness using the Olsen classification system. Grade 1 lesions are slightly palpable; grade 2 lesions are moderately thick, and grade 3 lesions are hyperkeratotic [2,12]. A recent study suggested that the Olsen classification is strongly associated with the risk of SCC development [13].

It is well known that a weakened immune system can reduce the ability to detect and eliminate cancer and precancerous cells, leading to the development of cancers (de novo or starting from precursor lesions). Among the reasons for immunosuppression are inherited and acquired diseases, medications, medical procedures, and several infections, such as the human immunodeficiency virus (HIV).

HIV infection increases the likelihood of developing certain cancers, such as Kaposi sarcoma and non-Hodgkin’s lymphoma, alone or in association with other infectious agents like the Epstein–Barr virus [14,15,16,17].

People living with HIV (PLWH) may also be especially susceptible to non-melanoma skin cancers (NMSCs), as well as other immunocompromised groups, such as solid organ transplant recipients (OTR) [18,19,20].

Despite the introduction of antiretroviral therapies, PLWH are at higher risk of developing non-acquired immunodeficiency syndrome (AIDS)-related cancers than the general population. Indeed, a study found that PLWH had a 2.6 times higher incidence of SCC compared to those without HIV [21]. This increased risk can be attributed to several factors, such as their longer life expectancy, accelerated aging, and impaired control of oncogenic infections caused by HIV-related immune suppression [15,21]. Although it has been shown that taking antiretroviral therapy reduces the risk of developing skin cancers compared to those who do not take it, PLWH still undergo chronic antigenic stimulation by the virus, suffering a state of chronic inflammation and cytokine dysregulation (even when HIV replication is suppressed and CD4 + T cell count is preserved) that can contribute to the development of lymphomas and other tumors [22,23].

Based on these factors, precancerous lesions of the skin, such as AKs, may put PLWH at greater risk of developing skin cancers than the immunocompetent population, as they do in transplanted patients [24]; therefore, the treatment of AKs in these immunosuppressed populations is crucial.

Usually, the treatment of AKs involves ablative procedures such as curettage, cryotherapy, topical photodynamic therapy (PDT), and topical chemotherapeutic or immune response modifier therapies. The former methods are preferentially reserved for isolated lesions, whereas topical therapies are preferred in cases with several lesions [2]. Topical treatments are intended to act on the so-called “field of cancerization”, an area of subclinical histological change located at the periphery of AK and detectable by histology or confocal microscopy [25,26]. From a clinical point of view, it is configured as a chronically photo-damaged area in which recurrent AKs and other signs of sun damage appear, such as wrinkles, solar elastosis, and telangiectasias.

In light of the clinic-pathological substrate underlying AKs in PLWH, the situation of multiple AKs in the context of a “field of cancerization” is more frequent in this type of patient, making the use of topical therapies preferable.

Regarding topical therapies, the most recent European Guidelines on diagnosis, treatment, and prevention of AKs mention 5-fluorouracil (5-FU), imiquimod, diclofenac, and tirbanibulin 1% ointment as effective modalities [2].

Tirbanibulin is a novel synthetic chemical entity that has shown potent antiproliferative and antitumoral effects by inducing cell-cycle arrest and, ultimately, apoptotic cell death [27]. These effects are attributed to tirbanibulin’s role as a microtubule and Src kinase inhibitor with potent antiproliferative activity against keratinocyte growth.

In UV-damaged skin, a cascade of events activates Src oncogene expression, increases Src kinase activity, and enhances the EGFR/Erk1/2 signaling pathway, increasing epithelial-to-mesenchymal transition (EMT) marker expression [28]. Elevated levels of Src have been linked to AKs and SCCs and play a role in both primary tumor growth and metastases.

In 2020 and 2021, the Food and Drug Administration (FDA) and the European Medicines Agency (EMA) approved tirbanibulin 1% ointment for treating Olsen grade 1 AKs of the scalp and face [29].

Tirbanibulin has a short application course, once daily for five consecutive days, on a maximum of 25 cm^2^ of the affected area. The most common adverse events are localized skin reactions at the application site. They range from mild to severe, including erythema, scaling, crusting, and swelling. These manifestations have been shown to resolve promptly with therapy discontinuation [29].

In two identically designed double-blind trials, complete clearance occurred in 44% (Trial 1) and in 54% (Trial 2) of the patients treated with tirbanibulin 1% ointment; partial clearance (≥75% reduction in the number of lesions) at day 57 occurred in 68% (Trial 1) and 76% (Trial 2) of the patients [29].

The most recent guidelines have widely discussed the treatment of AKs in immunosuppressed patients, with particular attention to transplant patients [2]. Regarding PLWH, the management of AKs has not been widely discussed and validated in the literature.

Furthermore, evidence on the efficacy and tolerability of AK treatment in PLWH, especially with modern topical chemotherapy drugs such as tirbanibulin 1% ointment, is limited.

Therefore, the present work aims to evaluate the response rate to the intervention with tirbanibulin 1% ointment for treating AKs in PLWH.

## 2. Materials and Methods

We retrospectively collected the clinical data of the PLWH who visited the Dermatology Department of the Policlinico Riuniti of Foggia, Italy, for skin cancer screening between 1 September 2023 and 30 September 2024.

Cancer screening in PLWH should align with the general population [30] and mainly concerns the prevention of carcinomas of the following types: colorectal, breast, prostate, and cervix. Since lung, liver, and anal cancers have a higher incidence and mortality in PLWH, screening for the early diagnosis of these tumors is also indicated [31]. Screening for skin cancers is generally recommended in PLWH, but national guidelines do not provide specific recommendations [32].

PLWH that attended the Infectious Disease Unit of the Policlinico Riuniti, Foggia, Italy, were invited to perform a skin cancer screening by their infectious disease specialist (S.L.C.). PLWH who consented to be screened for skin cancers were visited on the same day of the infectious disease visit in the Dermatology Unit of the same hospital by at least one dermatologist (G.C. or M.M.). The dermatologist performed a complete body examination using a handheld dermatoscope (DL5 dermoscope [DermLite, Aliso Viejo, CA, USA]) to observe skin lesions with tenfold magnification. In case of detection of a clinical/dermatoscopic suspicion of skin cancer, a plastic surgery visit was scheduled to perform a surgical excision of the suspected lesion. In the case of detection of at least one AK, the patient received a prescription for a topical therapy to treat AK and also the surrounding field of cancerization. Given the short application course and the high tolerability reported in previous studies [2,29], tirbanibulin 1% ointment (once daily, at night, for 5 consecutive days) was prescribed as a topical therapy for patients with single or multiple, non-hyperkeratotic, non-hypertrophic AKs (Olsen grade I) and moderately thickened, palpable AKs (Olsen grade II).

To assess the severity of AK/AKs, the actinic keratosis area and severity index (AKASI) score was calculated at the time of diagnosis using the calculator available on the website https://akasicalculator.com/it/#nav-onepage (accessed on 1 December 2024) [33].

The number of lesions at the first visit (time 0, T0) was also counted.

At the follow-up visit, 4–8 weeks after the end of treatment, the number of lesions was counted again and the AKASI score was calculated. In addition, the patients were asked to rate the severity of the skin reaction following the application of the tirbanibulin 1% ointment with a value ranging from 0 to 5 (0 = “no reaction”; 5 = “significant erythema, edema, desquamation, itching, pain”).

The response to treatment was considered satisfactory if the number of lesions found at follow-up was zero (complete healing) or reduced by more than 75% (partial healing) [29]; when the number of lesions did not reduce by ≥75% the patient was considered not healed.

In patients who consented, photographic documentation of the AKs was performed at the time of diagnosis (time 0) and after therapy (time 1).

This observational retrospective study adhered to the ethical standards of the institutional and national research committees, the 1964 Helsinki Declaration, and its later amendments. All the patients gave informed consent to participate in the study.

Categorical variables were expressed in absolute numbers and percentages, whereas continuous variables were expressed as mean and standard deviations (SD).

## 3. Results

During the study period, 90 PLWH consented to undergo skin cancer screening. Ten of these patients (11.1%) were found to have AKs and received topical therapy with tirbanibulin 1% ointment as first-line treatment. These patients have been retrospectively studied in detail.

They were all males with a mean age of 69.3 years (range 53–75 years, SD 5.8). In all cases, the diagnosis of HIV infection had been discovered more than 10 years earlier; on average, the diagnosis of HIV infection had been known for 27.2 years (range 12–39 years, SD 8.7). All the patients were regularly taking oral or injectable antiretroviral therapy prescribed by the infectious disease specialist. The HIV RNA load in plasma measured within the last month before the dermatological visit was undetectable in all patients (<20 copies/mL).

Six out of ten patients had extra-dermatological comorbidities (Table 1).

In addition, one patient was further immunosuppressed because he had undergone a liver transplantation 7 years earlier (following HCV-related cirrhosis), for which he was also taking oral tacrolimus therapy. This patient was the only one with a personal history of skin cancers (two basal cell carcinomas and one squamous cell carcinoma surgically removed in the previous 4 years).

The risk factors for developing AKs (Fitzpatrick skin phototype, sunburns during childhood, use of photosensitizing drugs) of the 10 studied patients have been summarized in Table 1. None of the patients were regular sunscreen users.

Two out of ten patients had already received treatments for AKs with topical drugs and liquid nitrogen cryotherapy in the previous two years at other hospital facilities.

At the first dermatological visit (T0), AKs were located in 9 out of 10 patients on the face and/or scalp. Only one patient had AKs exclusively on the back of the hands (Table 1).

On average, at T0, the number of lesions was 8.2 (range 1–19; DV 7.28) and the average AKASI score was 4.20 (range 1.2–8.8; DV 3.34). Most patients (7/10) had Olsen grade I and II AKs; three had only grade I AKs (Table 1).

At T0, two of the ten patients with AKs also had several skin lesions suspicious for skin cancers: histological examinations confirmed the clinical diagnoses of cutaneous melanoma and basal cell carcinoma in one patient and of basal cell carcinoma in another patient.

### Follow-Up After Treatment

On average, the follow-up visits were performed 18 weeks after the first visit (8–40 weeks, DV 11.75). Only two patients reported a mild inflammatory skin reaction (rated “1” on a scale of 0 to 5) to applying tirbanibulin 1% ointment, which resolved within 3–4 days. Eight patients did not experience any skin reactions at the site of the ointment application.

**Figure 1:**
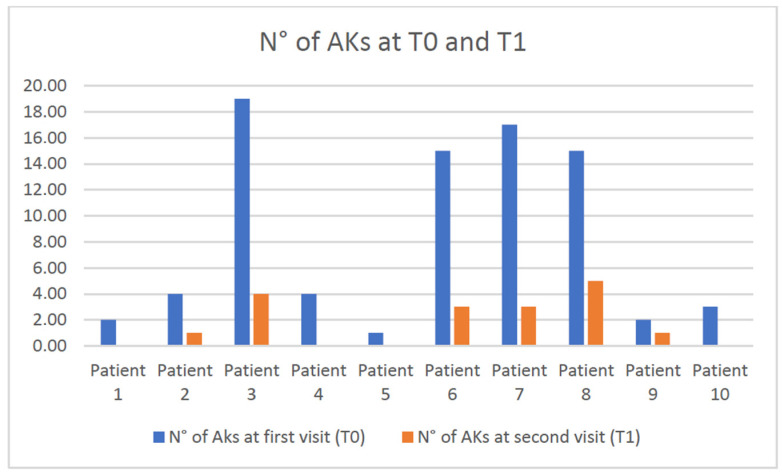
The actinic keratosis (AK) number before and after tirbanibulin 1% ointment treatment in the ten patients of our study.

At the follow-up visit (T1), the average number of AKs was 1.7 (range 0–5; DV 1.88) (Figure 1) and the average AKASI score was 1.5 (range 0–3.4; DV 1.7).

Based on the number of lesions, the response to treatment was considered satisfactory in 8 out of 10 patients who obtained a reduction in the number of lesions ≥75%; conversely, in 2 out of 10 patients, the tirbanibulin treatment did not determine a decrease in the number of lesions more significant than 75%. Therefore, they were not considered healed (Figure 1, patients n° 8 and 9). However, these patients presented at T1 a lesion number that had been reduced by at least 50% compared to T0 (from 15 to 5 lesions in case 8 and from 2 to 1 lesion in case 9).

**Figure 2:**
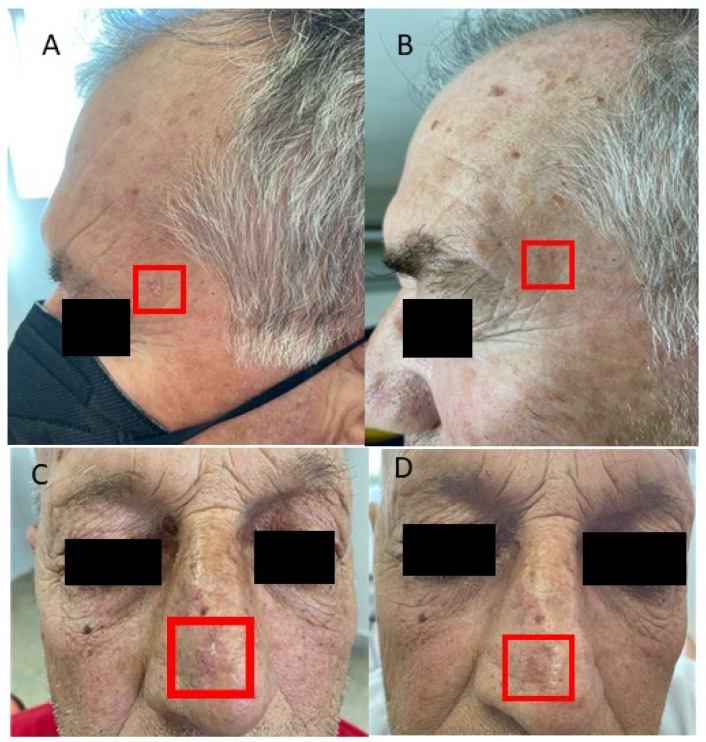
Actinic keratosis of the left temple before treatment with tirbanibulin 1% ointment (**A**) and complete resolution after therapy (**B**) in one patient; actinic keratosis of the nose before treatment (**C**) and complete resolution after therapy (**D**) in another patient.

**Figure 3:**
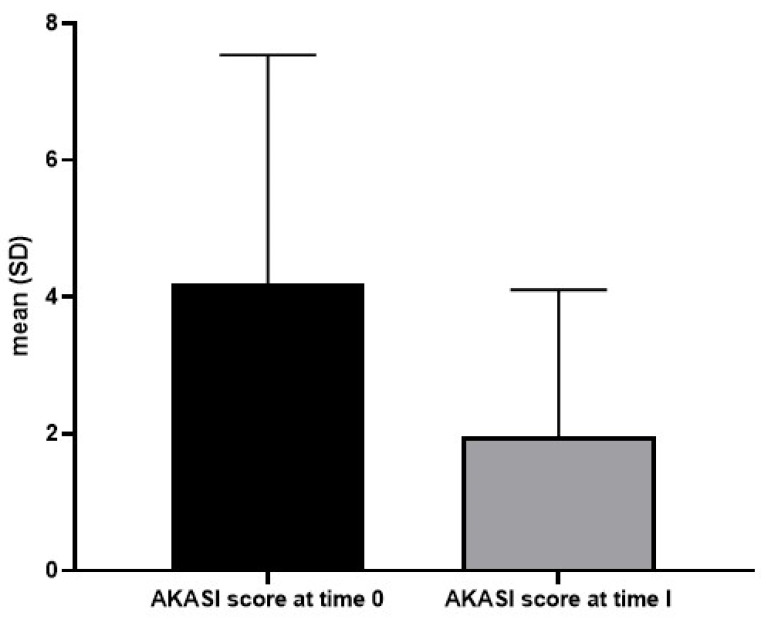
Mean actinic keratosis area and severity score (AKASI score) before and after treatment with tirbanibulin 1% ointment in the 10 patients of our study.

Figure 2 shows the pre- and post-treatment conditions in some AK patients treated with tirbanibulin 1% ointment; Figure 3 expresses the mean AKASI score before and after treatment.

## 4. Discussion

The results obtained in this study confirm the efficacy and tolerability of tirbanibulin 1% ointment in treating AKs. It is worth noting that the present work is the first to evaluate the efficacy of this drug in PLWH. Overall, in the present series, the AKASI score was significantly reduced after therapy: from a pre-treatment mean AKASI score of 4.20 ± 3.34 to a post-treatment mean AKASI score of 1.57 ± 1.7. Notably, tirbanibulin 1% ointment was well tolerated in all our patients, as none reported severe adverse reactions (grade 4–5 on a scale of 1 to 5). In our series, satisfactory responses were obtained in 8 out of 10 patients, according with a recent multicentric Italian study performed in a setting of immunocompetent patients [34]. Nazzaro et al. studied 250 patients affected by AKs recruited in 15 Italian dermatological clinics, including the Dermatology Unit of the Policlinico Riuniti of Foggia; treatment with tirbanibulin led to a satisfactory response in 88.8% of cases, and the response rates were higher (97%) when the follow-up was closer (8 weeks). Only seven (2.8%) grade 4 adverse events were recorded by Nazzaro et al., whereas we did not detect them in our smaller case series of PLWH [34]. The efficacy of tirbanibulin 1% ointment in treating skin field cancerization has recently been described in two immunosuppressed patients following solid organ transplantation [35]. These data support the positive outcome obtained in one of our patients who was immunosuppressed for pharmacological and infectious reasons (liver transplantation and HIV infection). Indeed, it is well known that chronically immunosuppressed patients, especially OTRs, have a higher risk of developing AK and SCC. The increase in skin cancer in OTRs is secondary to the long-term immunosuppressive therapies required for transplanted organ survival, which impairs the immune system’s ability to eliminate atypical cells. This increased risk in OTRs includes many types of cutaneous cancers and is exceptionally high for SCC, which some studies estimated to be 100-fold higher compared to the general population [36,37].

The most recent European guidelines on the diagnosis, treatment, and prevention of AKs provided ample space for managing AKs in immunosuppressed patients; however, they only referred to solid OTRs, and immunosuppression for other reasons, such as HIV infection, was not mentioned.

The authors underlined that immunosuppressed subjects frequently show multiple, metachronous evolving lesions over vast skin fields. Therefore, purely lesion-directed therapies may not be sufficient to obtain disease control. Furthermore, repetition of the treatment is often necessary due to a more aggressive disease course and a higher portion of treatment-resistant lesions in this population. The threshold to biopsy cutaneous and mucosal lesions to rule out progression to cutaneous SCC should be lower in immunosuppressed subjects than in immunocompetent individuals.

Based on randomized controlled trials and systematic reviews, the treatments that should be offered to immunocompromised patients with single and multiple AKs and a field of cancerization (grade of recommendation B) are conventional PDT, 5-FU 5% cream, and diclofenac sodium 3% in hyaluronic acid gel 2.5% [2]. A recent study on the treatment of AKs in OTRs found that the proportion of patients achieving 75% AKs clearance at 4 weeks post-treatment was 92% after 5-FU and 43% after imiquimod; at 8 weeks, the proportions were 75% after 5-FU and 43% after imiquimod. The response rate to the intervention with tirbanibulin 1% ointment found in our study was intermediate between the response rates showed in OTR treated with 5-FU at 4 and 8 weeks: indeed, 8 out of our 10 patients obtained a reduction of at least 75% of the AKs number after tirbanibulin treatment. Therefore, the response rate of the intervention with tirbanibulin was in line with that with 5-FU and more effective than that with imiquimod [38].

Cohort studies on the efficacy of tirbanibulin 1% ointment in treating AKs in immunocompromised patients are lacking.

Currently, tirbanibulin 1% ointment has been indicated for treating single or multiple AKs and the field cancerization of the face and scalp only in immunocompetent patients (grade of recommendation B, level of evidence 1) [2].

PLWH represents a unique subpopulation with sociodemographic features and dermatologic conditions different from the general population [39]. Paradoxically, the longevity achieved thanks to the use of antiretroviral therapy in PLWH has resulted in higher rates of cancers that prevail in older age or are induced by prolonged exposure to physical (skin cancer), chemical (lung, skin cancers), and biological (skin, anal, genital, liver cancers) mutagens [40].

Compared to immunocompetent subjects, PLWH have a more than two-fold higher incidence rate of keratinocyte carcinomas, especially SCC [41].

The systemic and cutaneous immune impairment induced by HIV has several different pathogenetic mechanisms that explain the link between this infection and skin cancers.

First, HIV shows a tropism for human immune system cells, such as macrophages, dendritic cells, and T lymphocytes (CD4 and CD8) [42]. Direct contact between CD4 T cells and HIV-pulsed dendritic antigen-presenting cells triggers the virus’s replication, leading to the death of both cell types. When CD4 T cells and antigen-presenting cells count decrease meaningfully, the skin becomes susceptible to opportunistic infections and neoplastic diseases.

In addition, HIV seems to activate proto-oncogenes, cause alterations in cell cycle regulation, and downregulate tumor suppressor genes, including p53 [43]. HIV could also determine microsatellite gene instability and genetic alterations, promoting the development of different cancers, including NMSCs. Finally, HIV infection may stimulate pro-angiogenesis signaling that could lead to endothelial abnormalities. These alterations could promote tumor growth and metastasis [42]. Skin cancers are the majority of neoplasias among PLWH, and NMSCs are the most frequent skin cancers in this population. NMSCs are usually more aggressive, as evidenced by an increased risk of metastatic disease and mortality compared to immunocompetent subjects. They tend to occur at a younger age, have an increased recurrence rate, and have an overall poorer outcome than the general population [42]. A low CD4 count (<200/microL) and high viral load (>10,000 copies/mL) in PLWH are associated with a greater risk of developing a primary NMSC and of having a recurrence [44]. Interestingly, an increased skin cancer risk has been documented not only among Caucasian PLWH but also among a South African Black population of PLWH [45]. On these grounds, PLWH, especially those with severe immunosuppression and those with prior NMSC (or both), should visit a dermatologist at least annually for a full-body skin check [42].

We are aware that the small number of patients included in the present series and the absence of a control group are the study’s limitations, which do not allow us to draw general conclusions. However, the present case series comprises patients from a specific population subgroup, namely PLWH. To date, very few reports in the literature are available on treating AKs in PLWH, and the present study represents a preliminary work that attempts to fill this knowledge gap.

## 5. Conclusions

Regrettably, studies on managing AKs in PLWH are scarce. Although our case series is limited to ten patients, our preliminary results suggest that PLWH on antiretroviral therapy may benefit from tirbanibulin 1% ointment therapy, presenting response rates similar to those of immunocompetent patients. Therefore, this treatment, which is the most manageable among the topical therapies suggested by the international guidelines since it requires only five consecutive days of application, might also be beneficial in contexts of immunodeficiency.

The treatment of precancerous lesions in PLWH needs further investigation. More extensive studies with longer follow-up times could further confirm these preliminary results.

## Figures and Tables

**Table 1:** Demographic and clinical features of the ten patients living with HIV affected by actinic keratoses.

Baseline Features of the Studied Patients	
Number of patients	10
Male sex	10/10
Mean age ± SD (years)	69.5 ± 5.8
Year from HIV diagnosis (years)	27.2 ± 8.7
**Fitzpatrick Phototype**	
II	8/10
III	2/10
History of sunburns in childhood	6/10
**Comorbidities**	
Hypertension	2/10
Hypercholesterolemia	3/10
Liver transplantation	1/10
HBV, HCV infection	1/10
Use of potentially photosensitizing drugs	1/10
History of non-melanoma skin cancers	1/10
Previous AKs	2/10
**Site of AKs**	
Only face	4/10
Only scalp	1/10
Face and scalp	3/10
Other sites	1/10
Face and other sites	1/10
**Olsen Grade of AKs at T0**	
I	3/10
I–II	7/10
II	0/10

## Data Availability

The data supporting this study’s findings are available from the corresponding author upon reasonable request.

## Abbreviations

The following abbreviations are used in this manuscript:

AK	Actinic keratosis
UV	Ultraviolet
HIV	Human immunodeficiency virus
PLWH	People living with HIV
SCC	Squamous cell carcinoma
NMSC	Non-melanoma skin cancer
HPV	Human papillomavirus
AIDS	Acquired immunodeficiency syndrome
FDA	Food and Drug Administration
AKASI	Actinic keratosis area and severity index
